# Analysis of Favorable Process Conditions for the Manufacturing of Thin-Wall Pieces of Mild Steel Obtained by Wire and Arc Additive Manufacturing (WAAM)

**DOI:** 10.3390/ma11081449

**Published:** 2018-08-16

**Authors:** José Luis Prado-Cerqueira, Ana María Camacho, José Luis Diéguez, Álvaro Rodríguez-Prieto, Ana María Aragón, Cinta Lorenzo-Martín, Ángel Yanguas-Gil

**Affiliations:** 1 Department of Manufacturing Engineering, Universidad Nacional de Educación a Distancia (UNED), 28040 Madrid, Spain; jprado28@alumno.uned.es (J.L.P.-C.); alvaro.rodriguez@invi.uned.es (Á.R.-P.); amaragon@invi.uned.es (A.M.A.); 2 Department of Design in Engineering, University of Vigo, C/Torrecedeira 86, 36208 Vigo (Pontevedra), Spain; jdieguez@uvigo.es; 3 Applied Materials Division, Argonne National Laboratory, 9700 Cass Ave, Lemont, IL 60439, USA; lorenzo-martin@anl.gov (C.L.-M.); ayg@anl.gov (Á.Y.-G.)

**Keywords:** additive manufacturing, WAAM, GMAW, cold metal transfer, hardness, mechanical properties, thermal input, microstructure

## Abstract

One of the challenges in additive manufacturing (AM) of metallic materials is to obtain workpieces free of defects with excellent physical, mechanical, and metallurgical properties. In wire and arc additive manufacturing (WAAM) the influences of process conditions on thermal history, microstructure and resultant mechanical and surface properties of parts must be analyzed. In this work, 3D metallic parts of mild steel wire (American Welding Society-AWS ER70S-6) are built with a WAAM process by depositing layers of material on a substrate of a S235 JR steel sheet of 3 mm thickness under different process conditions, using as welding process the gas metal arc welding (GMAW) with cold metal transfer (CMT) technology, combined with a positioning system such as a computer numerical controlled (CNC) milling machine. Considering the hardness profiles, the estimated ultimate tensile strengths (UTS) derived from the hardness measurements and the microstructure findings, it can be concluded that the most favorable process conditions are the ones provided by CMT, with homogeneous hardness profiles, good mechanical strengths in accordance to conditions defined by standard, and without formation of a decohesionated external layer; CMT Continuous is the optimal option as the mechanical properties are better than single CMT.

## 1. Introduction

One of the challenges in Additive Manufacturing (AM) of metallic materials is to obtain workpieces free of defects and with excellent physical, mechanical and metallurgical properties [1] to satisfy the strict requirements of engineering applications. Obtaining such mechanical requirements is a hard task, especially in parts fabricated as the result of layer by layer addition of the material. AM of metallic materials involves different techniques (powder bed fusion, binder jetting, sheet lamination, and directed energy deposition) and metals generally must be weldable and castable to be successfully processed in AM [2]. Till now, there have been only a limited number of commercial alloys used in AM [3], so there is a need to increase the number of alloys to be processed by AM techniques in order to widen the application fields. Most of the current commercial metallic materials for AM are steels [4,5,6,7], aluminum [8], and titanium alloys [9,10].

Wire and arc additive manufacturing (WAAM) is a wire-feed AM process and one of the most promising techniques for producing larger components with moderate complexity and relative low costs compared to other AM techniques for metals [11]. WAAM processes have a promising future. Designs are already being made using this technique in different fields where WAAM is performing very well; some of them are lightweight aerospace components (landing gear parts, wing ribs or stiffeners) [12], wind tunnel models [13], bridges [14] and/or complex constructive features (such as the dragon bench in the manufacture of furniture), that could not be made with conventional processes such as casting, or computer numerical controlled (CNC) milling, among others [15].

WAAM processes generally involve high residual stresses due to high deposition rates and heat inputs [16]. The influences of process conditions (for example, energy input, wire-feed rate [17], welding speed and/or deposition pattern [18]) on thermal history, microstructure and resultant mechanical and surface properties of parts need to be analyzed [16] as there is not enough knowledge in the scientific community yet.

As explained in the work by Ge et al. [4], during WAAM processes, the added layers of material suffer a complicated thermal history that includes, among others, melting, fast cooling, solidification, and/or partial remelting, that greatly influence the final properties of the parts produced by these techniques.

A recent study about the microstructure is the one from Wang et al. [19], where mechanical properties of thin-walled parts of the die steel H13 were also analyzed, showing that the tensile properties were anisotropic but could become isotropic after 830 °C of heat treatment (annealing) for 4 h. Yan et al. [20] studied the effect of temperature gradient, solidification velocity, and alloy composition on grain morphology in AM of metallic materials. In the overview article of Herzog et al. [21], special attention was paid in analyzing AM specific grain structures, resulting from the complex thermal cycle and high cooling rates. Kok et al. [22] highlighted that anisotropy and heterogeneity in the mechanical properties of metallic AM parts are mainly due to the anisotropy and heterogeneity of the microstructure and material properties. Another work investigating the relationship between the microstructure and mechanical properties of the titanium alloy Ti-6Al-4V fabricated by the WAAM process is reported by Wang et al. [23].

On the other hand, in the work from Szost et al. [24], porosity, microstructure, and micro hardness of Al-6.3%Cu samples fabricated by WAAM were investigated considering cold metal transfer (CMT) variants, pulsed CMT, and advanced CMT. A very interesting paper from Cong et al. [25] explores in depth the influence of the arc mode for different CMT variants (CMT, CMT pulse, CMT advanced, and CMT pulse advanced) on the porosity of an aluminum alloy fabricated by WAAM. In this work, CMT pulse advanced showed excellent performance in controlling porosity, being the most suitable variant for depositing aluminum alloys. Other defects such as lack of fusion, have been also analyzed by the same authors in their work of 2018 [26] for maraging steel.

Mechanical properties obtained by WAAM, including hardness, are also a promising field of study as shown in works from Horgar et al. [27], where AA5183 aluminum alloy wire was deposited on an AA6082-T6 plate as substrate. Wu et al. [28] investigated the influence of the molten pool size on the microstructure and mechanical properties of pieces of Ti-6Al-4V alloy, whereas Lewandowski and Seifi [29] presented a review of mechanical properties for the most common alloys used in AM of metals (Ti-6Al-4V, TiAl, stainless steel, Inconel 625/718, and Al-Si-10Mg).

Micro-geometrical properties such as roughness are also being investigated, as in the case of manufacturing of multi-layer single-pass thin-walled parts [30] and in the work of Li et al. [31].

Arrizubieta et al. [32] presented a novel manufacturing technique combining laser metal deposition, laser beam machining, and laser polishing processes for the manufacturing of a complex Inconel 718 part, resulting in a cleaner technique as conventional machining operations were eliminated. Another interesting work showing a suitable full-dimension sustainability life cycle assessment framework of parts produced by AM is the one presented by Ma et al. [33].

As we can see, due to the great variety of dimensions and parameters involved and their interdependencies [34], AM processes require multidisciplinary research, and many investigation lines are already open to reach a deeper knowledge about these technologies. In the present work, three-dimensional (3D) metallic parts of mild steel wire (AWS ER70S-6) are built with a WAAM process by depositing beads of weld metal layer by layer on a substrate of a S235 JR steel sheet of 3 mm thickness, using as the welding process gas metal arc welding (GMAW) [35] with Cold Metal Transfer technology [36], combined with a positioning system such as a CNC milling machine [37]. The paper will show some interesting results based on hardness measurements, along with complementary values of tensile strength at the working area and microstructure information.

## 2. Materials and Methods

### 2.1. Materials for WAAM process

Experiments have been carried out on a substrate of a S235JR steel sheet of 3 mm thickness, 150 mm long and 100 mm wide. This substrate has two main functions: as a support for the deposited metal and as a heat dissipation system for the heat generated during the process by conduction transfer through the aluminum work table.

The wire material (AWS ER70S-6) is a 0.8 mm diameter mild steel wire with a copper coating supplied on a 15 kg coil. This steel is commonly used in a lot of applications related to construction work, pipes, shafts, car bodies, tanks, steel castings or forgings, and general shop fabrications.

The properties of the base material (substrate) and the deposited material are shown in Table 1. The density of both materials is approximately the same, while the mechanical properties are better for the case of the deposited material.

Chemical composition of welding wire is shown in Table 2.

The results of the process depend on the protecting gas. It has been used a mixture composed of CO_2_ (15%) and Argon (85%) that led to the stability of the process, improvement in the surface finishing quality, and a reduction of the splatters. It has been observed that the welding drops are smaller with the reduction of the amount of CO_2_.

### 2.2. WAAM Equipment

**Figure 1:**
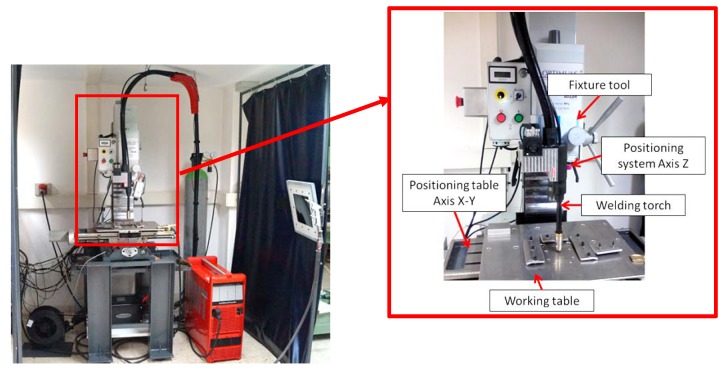
Setup of the integrated WAAM system in the positioning table.

The WAAM equipment is composed of two different systems (Figure 1), as described in detail in a previous work [38]:

• Welding system: cold metal transfer technology, patented by Fronius®, was used as welding process with a Fronius TPS 4000 CMT R machine (Fronius International GmbH, Wels, Austria). In this technology the intensity and voltage control is made during the deposition. By virtue of this principle, the temperature of welding temperature is reduced and the wire movement is optimized. As a result of this, the quality of weld beads is better than using conventional GMAW welding [39].

• Positioning system. The control of the movement in an easy way was made by a BF 30 Vario Optimum CNC milling machine (Optimum Maschinen Germany GmbH, Hallstadt, Germany). It has been adapted by fixing the welding torch to the milling head in the Z axis, while the X-Y table of the CNC system enables the deposition of a layer in the fixed Z level. To deposit the next layer, the Z axis elevates the torch and makes the deposition in the next Z level.

As shown in Figure 1, an auxiliary working table has been developed in order to isolate electrically both systems as well as to cool the working area.

### 2.3. Fabrication of Samples by WAAM

**Figure 2:**
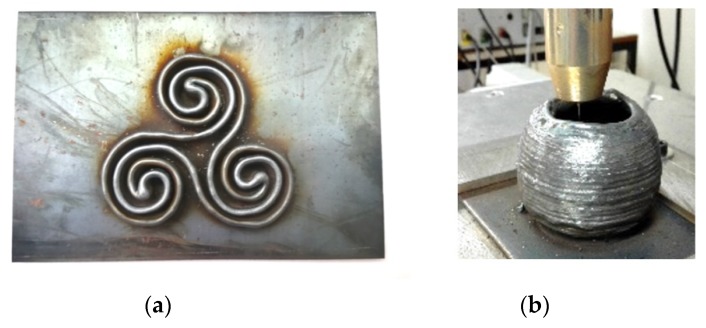
Examples of geometries obtained by WAAM: (**a**) Piece obtained by continuous trajectory and complex geometry in x-y direction; (**b**) Piece obtained by continuous trajectory and growing geometry in z direction.

As explained in a previous work [38], in WAAM processes, the final product is manufactured by melting a wire using an electric arc (Figure 2). The deposition of the material rate is much higher with respect to other metallic additive manufacturing methods. In addition, higher working speeds allow higher workload and a significantly lower price than with other methods [40].

In this work, a set of WAAM samples have been manufactured under different process conditions, considering the parameters with more influence in the mechanical properties of WAAM parts:

- WAAM process:
  ◦ Metal inert gas (MIG)
  ◦ CMT
  ◦ CMT Advanced polarity −5 (CMT Adv. pol. −5)
  ◦ CMT Advanced polarity 0 (CMT Adv. pol. 0)
  ◦ CMT Advanced polarity (CMT Adv. pol. +5)
  ◦ CMT continuous trajectory (CMT Cont.)
- Welding speed (constant = 400 mm/min)
- Deposition speed (constant = 2.5 m/min)
- Arc voltage (constant = 9.2 V)
- Current intensity (50 A, 66 A, 70 A, 78 A)
- Layer step (1.0 mm, 1.5 mm)

Different WAAM processes have been also applied to analyze the influence of using a conventional MIG process, a MIG process with CMT, and a MIG process with CMT advanced and different current polarities.

Cold metal transfer welding (CMT) is based on a MIG welding process but modified by a short-circuiting transfer process, firstly developed by Fronius Austria in 2004 [36]. CMT provides a controlled method of material deposition and low thermal input by incorporating an innovative wire feed system coupled with high-speed digital control [41]. With CMT, the arc only introduces any heat for a very brief period during the arc-burning phase and the arc remains stable, then CMT can be used everywhere and in every position [42].

The CMT advanced is an evolution of the previous process and it obtains a lower thermal input during welding with respect to the original CMT process thanks to the possibility of polarity change. This produces the reversal of the direction of the plasma jet several times per second leading to 35–40% lower thermal inputs [43]. The reversal of polarity takes place in the short-circuit phase so that this welding process guarantees the high stability expected from cold welding [44]. Thermal input is usually calculated based on the Equation (1):

(1) $$TI=\frac{V\cdot I\cdot \mu }{welding\;speed}$$

where *TI* is the thermal input in J/mm, *V* is the arc voltage in volts (V), *I* is the process intensity in Amperes (A), *µ* is the thermal efficiency that is a constant coefficient based upon the welding process used; finally, the welding speed is provided in mm/s.

The samples and the definition of the parameters used are presented in Table 3, including the calculation of thermal input.

**Figure 3:**
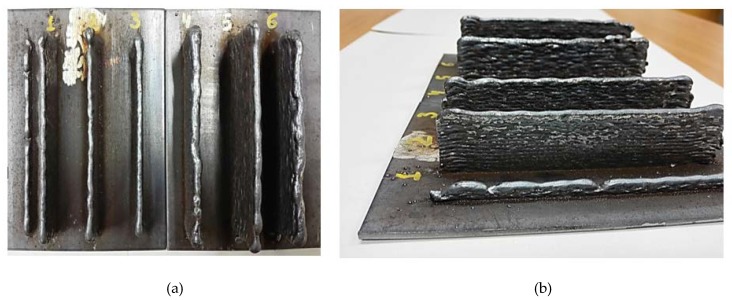
Manufacturing of samples nº 1 to 6: (**a**) Top view; (**b**) Lateral view.

Manufacturing of samples nº 1 to 6 is presented in Figure 3, where the substrate where they have been built is also shown.

**Figure 4:**
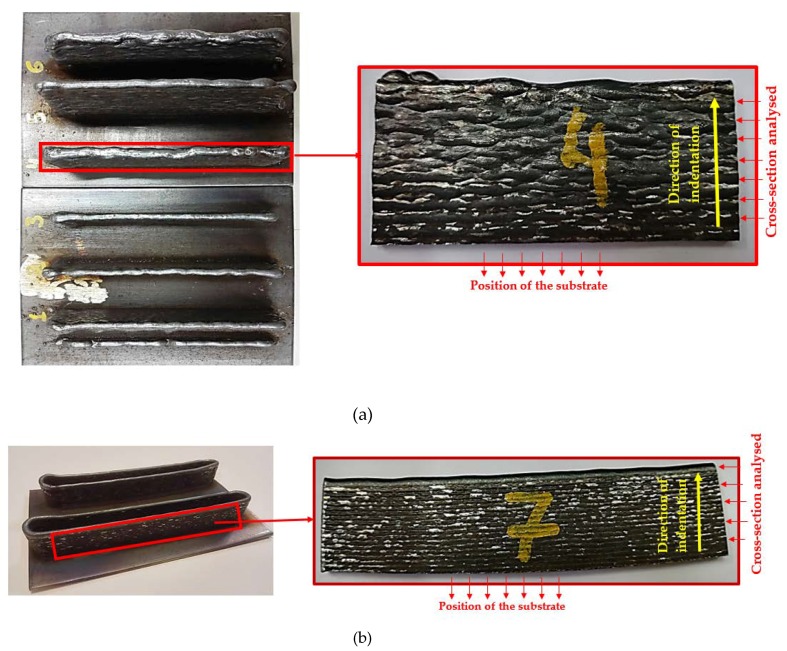
Location of the cross-section analyzed and the position of the substrate: (**a**) Samples nº 1 to 6, showing the location of the cross-section analyzed with sample nº 4; (**b**) Tool path during the deposition process in sample nº 7 and the final sample obtained.

Subsequently, the samples were removed from the substrate; an example of the final pieces obtained is shown in Figure 4a, with sample nº 4. Samples nº 2 and 7 share the same WAAM parameters; however, sample nº 7 differs from sample nº 2 in the way the wire is deposited. In order to provide a continuity during the deposition process, and to avoid edge effects, sample nº 7 has been obtained using a continuous tool path, as shown in Figure 4b (CMT continuous trajectory). Final sample after removing it from the substrate is also presented in this figure.

### 2.4. Brinell Hardness Tests

#### Brinell Hardness Tests and Measurement of the Ball Prints

**Figure 5:**
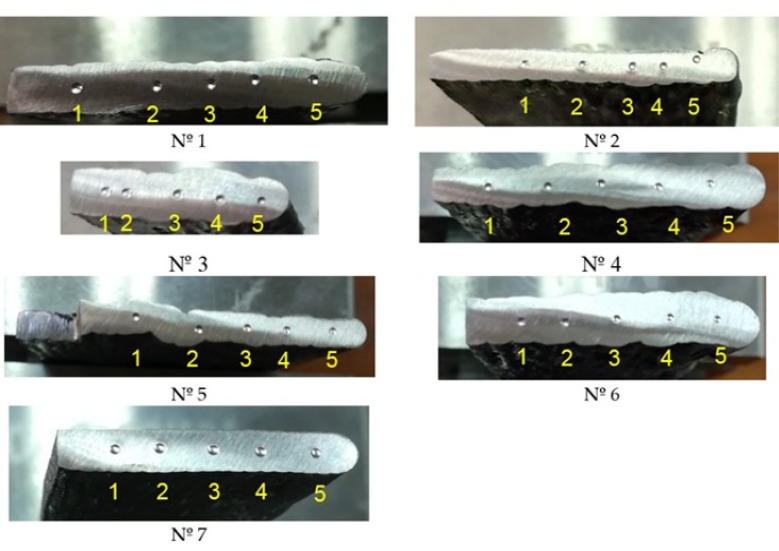
Brinell hardness tests applied to WAAM samples and identification of indentation points.

Brinell hardness tests have been developed [45] using a ball indenter of ϕ2.5 mm and a test force of 612.9 N. The ball prints imprinted at the surface of the seven samples are presented in Figure 5. A set of 5 points have been imprinted at the cross-sections analyzed, as explained in Figure 4a,b. These surfaces have been previously polished to obtain a smooth condition and free from oxides and lubricants. The numbering of the points increases from the location of the substrate (see yellow arrow in Figure 4a,b). The aim is to obtain a hardness profile for each sample to compare the observed behavior depending on the manufacturing parameters used in each case.

The measurement device to determine experimentally the print diameter is a profile projector TESA VISIO (TESA SA, Renens, Switzerland). Two indentation diameters measured at 90° have been obtained for each sample allowing to calculate a mean diameter of the indentation.

The Brinell hardness is proportional to the quotient obtained by dividing the test force by the surface area of the indentation left in the surface after removal of the test force.

The dispersion among measurements can be quantified using the reproducibility limit, *R*, which is calculated as shown in Equation (2) [46]:

(2) $$R=\frac{d_{max}-d_{min}}{\langle d\rangle }$$

where *d_max_* and *d_min_* are the largest and smallest diameters and 〈*d*〉 is the mean of measured diameters.

### 2.5. Determination of Mechanical Strength

Hardness is usually defined as resistance to permanent indentation. This testing provides a measurement of the material strength through its resistance to scratching. Thus, the possibility to predict tensile strength based on values of materials hardness is often used. Equation (3) provides the general relationship between hardness and tensile strength:

(3) UTS=k·H

where *UTS* is the ultimate tensile strength in MPa, *H* the hardness in a known scale and *k* is a coefficient. Several standards provide a correlation between hardness and tensile strength in steels using tables, charts, and coefficients of calculation, some of them are ASTM A370 [47], ISO 18265 [46], SAE J417 [48], being the ASTM standard the most consolidated and used.

### 2.6. Equipment and Measurement of Microstructure

Microstructural analysis has been performed using the following equipment from the center for nanoscale materials (CNM) of the Argonne National Laboratory: a high resolution and high vacuum, scanning electronic microscopy Hitachi S-4700-II (Hitachi, Krefeld, Germany)—equipped with electron dispersive spectroscopy (EDS) detector Bruker XFlash 6160 (Bruker, Billerica-MA, USA). The testing conditions were 10 keV and 10 mA.

## 3. Results

### 3.1. Evaluation of Hardness Profiles

As the WAAM process is layer-based, the most critical area is the one located at the overlapping of the two layers, or as close as possible to it. For this reason, we have chosen the positions of the indentations (ID), making sure that for each sample we have most of the points located at these critical areas. In Appendix A, the position of the points for every sample is shown. As we can see, most of the indentations are located following this criterion.

Table 4 provides the mean values of Brinell hardness along with the thermal input and the calculation of reproducibility limit (*R*) according to Equation (2), using the diameters of indentations.

Conventional MIG (sample 1) process provides the biggest thermal input and hardness along with the minimum *R* value (0.003). The CMT process (samples 2 and 7) with the lowest thermal inputs, provides an adequate dispersion among values, exhibited by their R values (0.064 and 0.025, respectively). Hardness values in samples 3 to 6 (CMT advanced) do not seem to follow a pattern dependent on thermal input, but hardness and thermal inputs adopt intermediate values.

**Figure 6:**
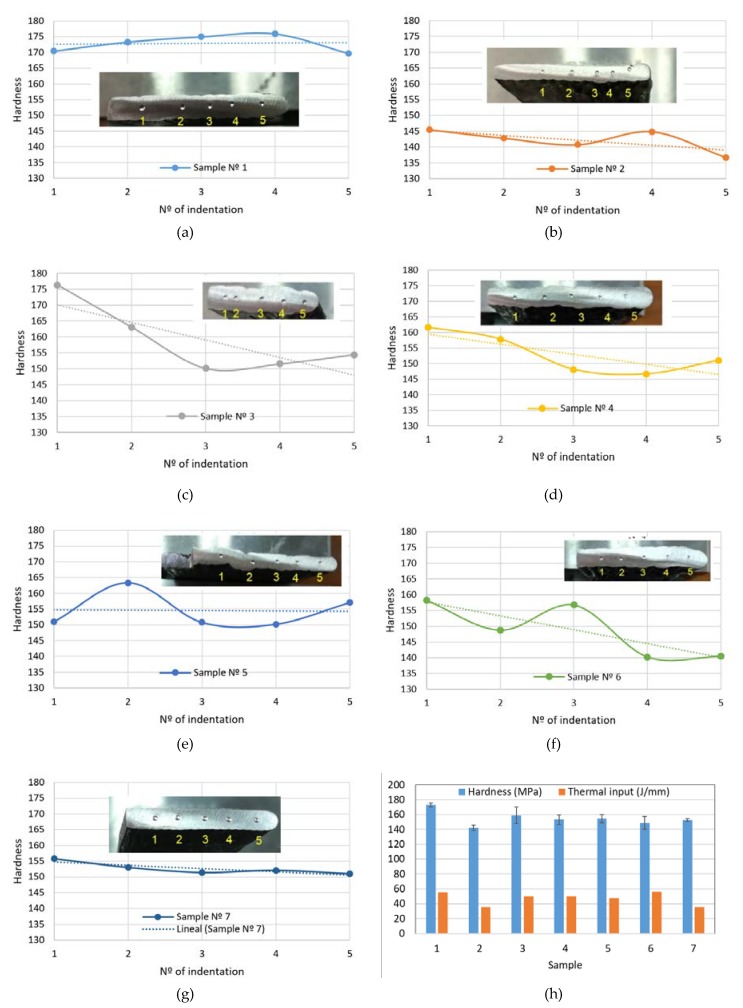
Brinell hardness profiles for the WAAM samples: (**a**) Sample nº 1, MIG (conventional); (**b**) Sample nº 2, CMT process; (**c**) Sample nº 3, CMT Adv. pol. 0; (**d**) Sample nº 4, CMT Adv. pol. 0; (**e**) Sample nº 5, CMT Adv. pol. −5; (**f**) Sample nº 6, CMT Adv. pol. +5; (**g**) Sample nº 7, CMT; (**h**) Mean hardness values with standard deviations and thermal inputs.

The hardness profiles are presented in Figure 6 along with the average values. A homogenous hardness profile is desirable as this means that the mechanical properties obtained by the WAAM process are appropriate and the in-service behavior of the parts is expected to be better than with the non-homogeneous ones.

As we can see in Figure 6h, according to the standard deviations of the hardness values, samples 3 and 6 present the least homogenous hardness profile, whereas samples 1 and 7 are the best ones.

### 3.2. Evaluation of Mechanical Strength

Using the hardness measurements, estimated ultimate tensile strengths (UTS) have been calculated and provided in Table 5.

As it was previously mentioned, the welding wire is an ER70S-6 type, described by the american society of mechanical engineers ASME SFA 5.18 standard [49], which indicates some recommended base materials to be welded using this type of welding wire; these are SA-36 [50], equivalent to S235JR, SA-285 [51], SA-515 [52], and SA-516 [53]. Table 6 exhibits the specified range of UTS for these materials. These values are used to help analyze the ultimate tensile strength (UTS) calculated using the hardness measurement performed in the 7 samples.

### 3.3. Microstructure Findings

**Figure 7:**
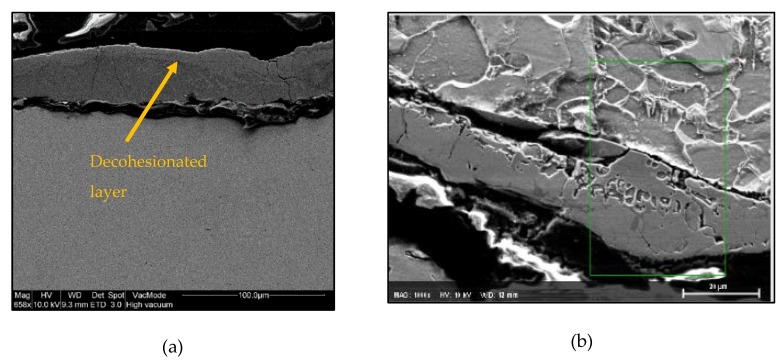
Scanning electronic microscopy (SEM) at the surface in Sample nº 1 (MIG conventional). (**a**) Decohesionated layer found; (**b**) Layer SEM image at 20 µm of scale.

Microstructural analysis of each sample has been performed using a high-resolution scanning electronic microscopy at the center for nanoscale materials (CNM) of Argonne National Laboratory. Figure 7a provides an image of the surface along the deposition direction in Sample nº 1 (MIG conventional); a decohesionated layer in the upper edge is observed in this sample (a zoom of this area is presented in Figure 7b, showing the microstructure more in detail). Appendix B shows the surface of deposited material along the thickness for samples nº 2 to 7, where no decohesionated layer is found.

Table 7 provides the compositional microanalysis of this layer observed in sample nº 1.

The external layer of sample 1 (MIG process) seems to be formed by Fe_3_C (6.67% C) and probably other complex carbides made up of some of the rest of the elements oxidized but present in the normal weight percentage according to the composition provided by the manufacturer (Mn 1.40–1.85%, Si 0.80–1.15%, Cu < 0.5%). In addition, in the process magnetite (Fe_3_O_4_) seems to be also present. Anyway, the external layer is a pernicious effect that could be avoided using the CMT process, as it is possible to see through the figure in Appendix B.

The presence of a decohesion layer implies poor surface properties; according to the analysis carried out, this layer comprises the formation of carbides (which are typically hard and brittle compounds) and oxides, which give the surface a poor surface finish and low resistance to external chemical and mechanical agents; therefore, these types of layers are not desirable and should be avoided.

**Figure 8:**
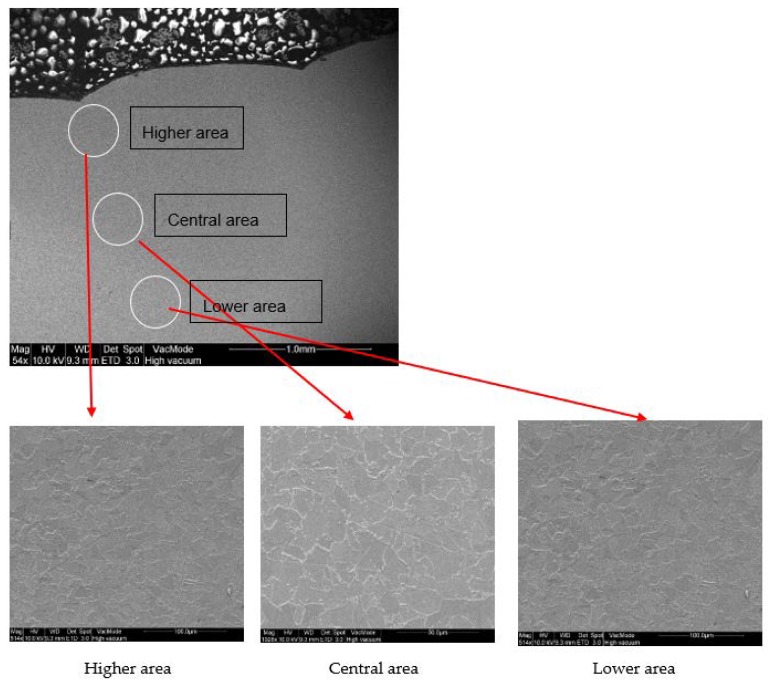
SEM Micrographs at interface between layers in Sample nº 1 (MIG conventional).

Additionally, micrographs at the interface between layers have been obtained to zoom into this area at the microstructural level (Figure 8). No special findings are found at this level.

## 4. Discussion

As indicated, a homogenous hardness profile is desirable as this means that the mechanical properties obtained by the WAAM process lead to better in-service behavior of parts than with non-homogeneous ones.

The most homogeneous profiles are obtained in samples numbers 1, 2, and 7 (in sample 2 the measurement of point 5 has been obviated as the print is too close to the surface). Homogeneous profiles for MIG procedure (sample 1) were also obtained in the work by Wang et al. [19]. Samples 2 and 7 present lower values of hardness than sample 1 (Table 4); this can be explained as the CMT process applies lower thermal inputs compared to the conventional MIG process and therefore, the sample 1 experience greater sub-cooling from the melting state and then, a microstructure of finer grains is expected. Bigger grain sizes at the microstructure lead to lower hardness values as grains limits contribute to block the movement of material dislocations.

Slight differences between sample 2 (CMT) and 7 (CMT Cont.) are due to the effect of the continuous path applied in sample 7 that, for the same thermal input due to the same process parameters, implies an accumulation of heat at the zone due to lower heat transmission and consequently, induces a higher thermal input than the one computed and, as explained before, this leads to a higher hardness value in sample 7.

Samples fabricated by CMT Advanced processes have a pronounced decreasing trend of the hardness profile, showing the highest values closer to the substrate (see Figure 6c–f). This is due to the chilling effect of the substrate that generates a higher cooling rate and therefore, the sub-cooling effect from the melting state is higher in this zone [5]. The results are in good agreement with the ones presented by Liberini et al. in their work from 2017 [54]; where an increase of hardness is also found close to the free surface as a result of the thermal chilling due to contact with the air at room temperature. In this work [54], the authors also stated that the cooling curve is the factor that most influences the final microstructure and that no important differences between the samples are obtained from different process parameters. With CMT Advanced, the mean hardness values are very similar for samples 3 to 6, and the thermal inputs as well.

The most inhomogeneous profiles are obtained in samples 5 and 6, where some peaks are observed. In these two cases a polarity of 5 and +5, respectively, is applied during the process, and the intensity applied is also different in both cases (66 and 78 A, respectively). However, regardless the different conditions, the mean hardness values are close between them and to the ones obtained with polarity 0. In general, we can conclude that the CMT Advanced process does not show a better performance of the process regarding the homogeneity of the hardness profile of the parts and the mechanical properties.

Indentation points are located at the overlapping area (or as close as possible) of the two layers, the most critical area for an additive manufacturing process. No significant influence of the position of the indentation points on the hardness values is observed at this level of analysis, being these results are in good agreement with those obtained by other authors using a similar methodology, such as Xu et al. [4]. Micrographs at the interface between layers did not show special findings at the microstructural level. Additional research will be conducted in future works in order to analyze locally the behavior between layers, combining higher resolution hardness tests and metallographic analysis in this area.

As WAAM is a layer-by-layer manufacturing process that uses a welding wire that melts on a previously welded substrate, it is important to ensure that the requirements of weldability, such as the mechanical properties of a material that are joined using the welding wire, are well suited. Using the recommendations provided by the Kobe Welding Handbook [55], the base material should present a minimum UTS between 400–480 MPa. Therefore, considering the requirements indicated in Table 6, in this evaluation, a range between 400 and 550 is considered suitable. Values higher than 550 MPa could lead to the appearance of hardness peaks between layers, which are not recommended as they do not guarantee the homogeneity of the mechanical behavior. This supposes that the estimated UTS at the surface of sample 1 (MIG conventional process), equal to 581.28 MPa, is greater than the upper limit that the new substrate should exhibit. The remaining mean values (samples 2 to 7) are between 400 and 550 MPa, nevertheless some specific values are above the upper limit (550 MPa) in samples 3 to 5. Thus, it can be concluded that CMT process (samples 2 and 7) and CMT Adv. pol. +5 (sample 6) provides the most adequate UTS values.

In addition, the microstructural analysis of each sample (1 to 7) has been performed using high-resolution scanning electronic microscopy. Homogeneity has been observed in the transition between layers in all samples. Nevertheless, a decohesionated layer in the upper edge is observed in sample 1 (MIG conventional). The external layer is a pernicious effect that can be avoided using the CMT process.

In agreement with other authors, there are no significant differences between the samples processed with different process parameters when using a particular WAAM process [16,54].

## 5. Conclusions and Future Work

After discussing the main results, Table 8 shows a summary of the best process conditions considered in this work.

Taking into account the hardness profiles and mean values, the estimated UTS derived from the hardness measurements and the microstructure findings, it can be concluded that the best process conditions are the ones provided by simple CMT, with homogeneous hardness profiles, good mechanical strengths in accordance to conditions defined by standard, and without formation of a decohesionated external layer; CMT Continuous is the optimal option as the mechanical properties are better than with single CMT (Figure 6h and Table 5).

In this study, we have been interested in defining global trends of the hardness at a macroscopic level for common values of manufacturing parameters to obtain results with a broad level of generality from an applicative point of view, of interest for users of these technologies. As there is still an important lack of information about the influence of the microstructure in the behavior of parts obtained by additive manufacturing processes in general, and in WAAM in particular, future work will be focused on developing an in-depth analysis about this promising topic, drawing special attention to the microstructure between layers, which is the most critical area in AM parts, and using higher resolution tests.

## Figures and Tables

**Table 1:** Properties of the substrate and the welding wire.

Mechanical Properties	S235 JR	AWS ER70S-6
Density (kg/m^3^)	7800	7833
Yield point (MPa)	235	420
UTS (MPa)	370–510	500–640

**Table 2:** Chemical composition of welding wire.

Element	C	Mn	S	Ni	V	Cr	Cu	Si	P	Mo
**wt%**	0.06–0.15	1.40–1.85	0.035 max	0.15 max	0.03 max	0.15	0.50	0.80–1.15	0.025	0.15 max

**Table 3:** Definition of parameters used for each sample and results.

Nº	Process	Intensity (A)	Thermal Input * ^1^ (J/mm)	Welding Speed (mm/min)	Deposition Speed (m/min)	Wall Thickness (mm)	Layer Step (mm)	Total Height (mm)	Layer Height (mm)
1	MIG	50	55.19	400	2.5	3.8	1.0	27.0	0.90
2	CMT	50	35.87	400	2.5	3.7	1.0	30.7	1.02
3	CMT Adv. pol. 0	70	50.22	400	2.5	4.2	1.0	20.2	1.44
4	CMT Adv. pol. 0	70	50.22	400	2.5	5.5	1.5	35.5	0.92
5	CMT Adv. pol. −5	66	47.36	400	2.5	4.5	1.5	41.2	1.07
6	CMT Adv. pol. +5	78	55.97	400	2.5	6.6	1.5	33.4	0.80
7	CMT Cont.	50	35.87	400	2.5	3.1	1.0	30.5	1.02

^1^ Note 1*: thermal input has been calculated based on the power (*V·I*) provided by the equipment, the welding speed and the thermal efficiency coefficients, typically *µ* (MIG) = 0.8, and *µ* (CMT) = 0.52 considering a 35% of lower thermal efficiency compared to MIG process [43].

**Table 4:** Process, Brinell hardness, and *R* values.

Sample nº	Process	Thermal Input (J/mm)	Brinell Hardness (Mean Value)	〈*d*〉	*d_max_*	*d_min_*	*R*
1	MIG	55.19	172.89	0.729	0.731	0.7285	0.003
2	CMT	35.87	142.14	0.790	0.809	0.759	0.064
3	CMT Adv. pol. 0	50.22	159.03	0.761	0.790	0.725	0.086
4	CMT Adv. pol. 0	50.22	153.00	0.778	0.799	0.759	0.051
5	CMT Adv. pol. −5	47.36	154.49	0.773	0.789	0.759	0.038
6	CMT Adv. pol. +5	55.97	148.84	0.792	0.815	0.771	0.056
7	CMT Cont.	35.87	152.67	0.772	0.784	0.765	0.025

**Table 5:** Estimation of Ultimate Tensile Strength values based on ASTM A370 [47].

UTS (MPa) Correlation per Indentation (ID) According to Figure 6.							
	Process						
ID	MIG	CMT	CMT Adv. pol. 0	CMT Adv. pol. 0	CMT Adv. pol. −5	CMT Adv. pol. +5	CMT-Cont.
**1**	573.33	479.99	516.38	498.34	498.39	467.42	528.44
**2**	583.30	473.06	499.98	483.64	562.20	465.82	505.24
**3**	587.47	468.90	495.17	488.64	497.70	534.68	499.66
**4**	589.84	477.92	561.66	541.73	495.74	490.68	501.99
**5**	572.46	454.32	590.94	558.59	537.12	544.38	498.00
**Mean**	581.28	470.84	532.82	514.19	518.23	500.59	506.67

**Table 6:** Ultimate Tensile Strength of typical base materials welded with ER70S-6 according to SFA 5.18 [49].

Base Material Specification	UTS (MPa)
SA-36 (equivalent to S235JR)	400–550
SA-285	310–515
SA-515	415–485
SA-516	380–485

**Table 7:** Microanalysis of decohesionated external layer observed in Sample 1 (MIG conventional process).

Element	Mn	C	O	Si	Cu	Fe
wt%	1.58	7.59	1.79	0.83	0.44	87.77

**Table 8:** Summary of the best process conditions according to homogeneous hardness profiles and mean values, the estimated ultimate tensile strengths (UTS) derived from hardness measurements, and the absence of the decohesionated layer at the surface found at microstructural level.

Sample nº	Process	Most Homogeneous Hardness Profiles (Figure 6a–g)	Highest Values of Mean Hardness (Figure 6h)	Best Estimated UTS [55] (Table 5)	Absence of Decohesionated Layer at the Surface (Figure A2)
1	MIG	X	X		
**2**	CMT	X		X	X
3	CMT Adv pol. 0				X
4	CMT Adv pol. 0				X
5	CMT Adv pol. −5				X
6	CMT Adv pol. +5				X
**7**	CMT Cont.	X		X	X

**Figure A1:**
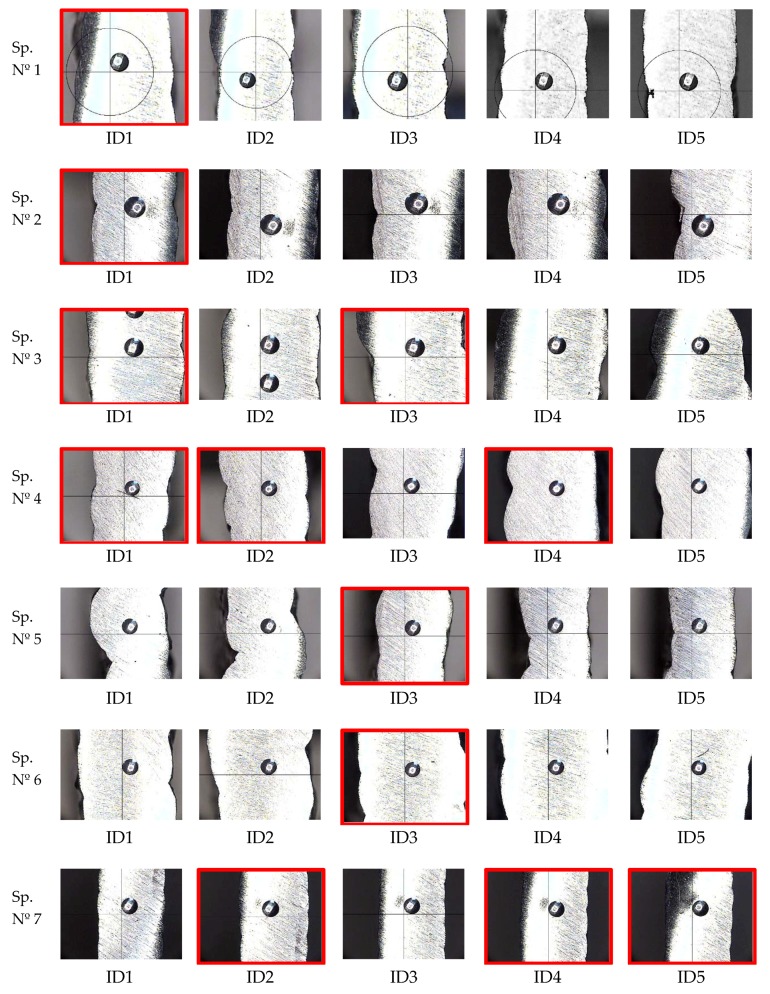
Brinell hardness tests applied to WAAM samples and identification of indentation points; images with red frame showing points located more clearly between layers.

**Figure A2:**
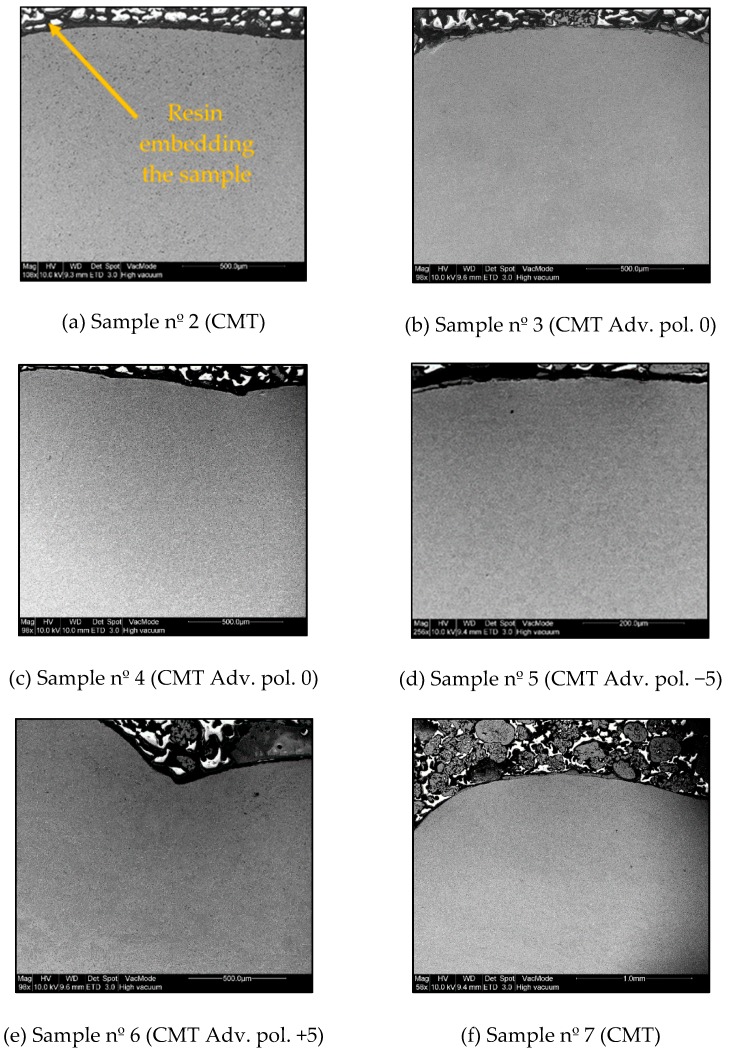
SEM images showing no decohesionated layer formation in the upper edge of the surface. (**a**) Sample nº 2, CMT process; (**b**) Sample nº 3, CMT Adv. pol. 0; (**c**) Sample nº 4, CMT Adv. pol. 0; (**d**) Sample nº 5, CMT Adv. pol. −5; (**e**) Sample nº 6, CMT Adv. pol. +5; (**f**) Sample nº 7, CMT Cont.

## Appendix A. Position of the Indentations

In Figure A1 the position of the indentation points for every sample is shown. Images of points located more clearly between layers are highlighted with red frame.

## Appendix B. Scanning Electronic Microscopy (SEM) to Detect Decohesionated Layer in the Upper Edge of the Surface

Figure A2 shows the surface of deposited material along the thickness for samples nº 2 to 7. The resin embedding the sample has not to be confused with the formation of the decohesionated layer that was only found in Sample nº1, using MIG conventional without CMT.
